# Metatranscriptomic Analysis Reveals Active Bacterial Communities in Diabetic Foot Infections

**DOI:** 10.3389/fmicb.2020.01688

**Published:** 2020-07-22

**Authors:** Fatemah Sadeghpour Heravi, Martha Zakrzewski, Karen Vickery, Matthew Malone, Honghua Hu

**Affiliations:** ^1^Surgical Infection Research Group, Faculty of Medicine and Health Sciences, Macquarie University, Sydney, NSW, Australia; ^2^QIMR Berghofer Medical Research Institute, Brisbane, QLD, Australia; ^3^Infectious Diseases and Microbiology, School of Medicine, Western Sydney University, Sydney, NSW, Australia; ^4^Liverpool Hospital, South Western Sydney LHD, Sydney, NSW, Australia; ^5^Liverpool Diabetes Collaborative Research Unit, Ingham Institute for Applied Medical Research, Sydney, NSW, Australia

**Keywords:** diabetic foot infection, RNA sequencing, metatranscriptomics, active bacterial community, resistome, virulence factors

## Abstract

Despite the extended view of the composition of diabetic foot infections (DFIs), little is known about which transcriptionally active bacterial communities are pertinent to infection, and if any differences are associated with increased infection severity. We applied a RNA sequencing approach to analyze the composition, function, and pathogenicity of the active bacterial communities in DFIs. Taxonomic profiling of bacterial transcripts revealed the presence of 14 bacterial phyla in DFIs. The abundance of the *Spiroplasma*, *Vibrio*, and *Mycoplasma* were significantly different in different infection severities (*P* < 0.05). Mild and severe stages of infections were dominated by *Staphylococcus aureus* and *Porphyromonas asaccharolytica*, respectively. A total of 132 metabolic pathways were identified of which ribosome and thiamin being among the most highly transcribed pathways. Moreover, a total of 131 antibiotic resistance genes, primarily involved in the multidrug efflux pumps/exporters, were identified. Furthermore, iron acquisition systems (synthesize and regulation of siderophores) and pathways involved in the synthesis and regulation of cell-surface components associated with adhesion, colonization, and movement of bacterial cells were the most common virulence factors. These virulence factors may help bacteria compete for scares resources and survive the host wound proteases. Characterization of transcriptionally active bacterial communities can help to provide an understanding of the role of key pathogens in the development of DFIs. Such information can be clinically useful allowing replacement of DFIs empirical therapy with targeted treatment.

## Introduction

Diabetic foot infections (DFIs) are a frequent cause of hospitalization and typically precede events such as lower extremity amputation (Lavery et al., 2003). Traditional approaches to identify pathogens colonized in DFIs have relied on culture-based methods that are limited to detect bacterial species grown under standard laboratory conditions (Heravi et al., 2020).

Over the last decade, several studies have identified that diabetic foot ulcers were composed of a complex bacterial community consisting of aerobes, anaerobes, fastidious, and unculturable microorganisms using 16S rRNA sequencing (Smith et al., 2016; Gardiner et al., 2017). The 16S rRNA approach is limited to genomically present but transcriptionally inactive bacterial communities. It also does not provide insight into the potential or actual function of the bacterial community in DFIs.

High-throughput RNA sequencing or metatranscriptomic analysis is a promising tool to obtain insights into the functionality of active bacterial communities. Metatranscriptomics has also addressed the limitation of microarray assays such as unspecific hybridization signals (Zhao et al., 2016).

The composition and function of the active bacterial communities in DFIs can provide a clue for understanding the actual role of microorganisms in infection progression and the improvement of therapeutic approaches. To the best of our knowledge, this is the first study applying an RNA sequencing approach to explore the composition, function, and virulence factors of the transcriptionally active bacteria in DFIs.

## Materials and Methods

### Ethics Statement

This study was approved by the South West Sydney Local Health District Research and Ethics Committee (HREC/14/LPOOL/487, SSA/14/LPOOL/489) and Macquarie University Human Ethics Committee (Reference No. 5201500839). All the experiments were performed in accordance with relevant guidelines and regulations. Informed consent has been obtained for this study.

### Patient Population

In this prospective study, 43 consecutive patients aged over 18 years presenting to the Liverpool Hospital High-Risk Foot Service with a clinically diagnosed DFI were enrolled over a 6-month period. Infection severity was determined using the International Working Group of the Diabetic Foot (IWGDF), Perfusion, Extent, Depth, Infection and Sensation (PEDIS) classification system, and patients were assigned accordingly (PEDIS 2 refers to mild infection, PEDIS 3 refers to moderate infection, PEDIS 4 refers to severe infection) (Lipsky et al., 2016). Since RNA sequencing approach requires RNA with high quality and integrity, after RNA extraction from all of the clinical samples and initial assessment, 16 samples that had high-quality and integrity RNA were selected for RNA sequencing.

### Sample Collection

After the DFI ulcer was cleaned with sterile 0.9% NaCl, a sterile single-use punch with a circular hollow blade was rotated around the affected area, and then the sharp debridement was collected by disposable forceps and preserved immediately in a 2 ml RNA*later* stabilization solution (Thermo Fisher Scientific, Waltham, MA, United States) for 24 h at 4°C and then stored at −80°C until processed.

### Sample Pretreatment

Infected tissue specimens (<25 mg) were homogenized in 1 ml TRIzol reagent (Invitrogen, Carlsbad, CA, United States) using TissueRuptor II (Qiagen, Hilden, Germany) at 230 V, 50/60 Hz for 10 s.

### RNA Extraction

The above pretreated tissue samples (<25 mg) were incubated in 1 ml TRIzol reagent for 5 min prior to further homogenization using 0.1 and 0.5 mm beads in a FastPrep-24 instrument (MP Biomedicals, Irvine, CA, United States) with a velocity of 5 m/s for 1 min while sitting on dry ice to break the bacterial cell wall. TRIzol^®^ Plus RNA Purification Kit (Invitrogen, Carlsbad, CA, United States) was used to extract high-quality RNA from homogenized tissue samples. Extracted RNA was further treated with TURBO DNase (Invitrogen, Carlsbad, CA, United States) and purified by AMPure XP beads (Beckman Coulter Life Sciences, San Jose, CA, United States) according to the manufacturer’s instructions.

### RNA Integrity, Synthesis of cDNA, and Illumina Sequencing

Quality and integrity of extracted RNA were evaluated in an Agilent 2100 Bioanalyzer system using microfluidics-based electrophoresis on microfluidic chips (Agilent Technologies, Santa Clara, CA, United States) which produced an RNA integrity number (RIN) as an output. Illumina whole transcriptome library preparation with rRNA depletion was performed using the Illumina Ribo-Zero Gold Epidemiology kit (Illumina Inc., San Diego, CA, United States) on 16 selected high-quality RNA samples. The RNA sequencing was run on one Illumina NovaSeq 6000 S4 flow cell (300 Cycle) by the Australian Genome Research Facility to achieve >150 million pair-end reads of 150 nucleotides per sample.

### Processing of Metatranscriptome Data

**FIGURE 1:**
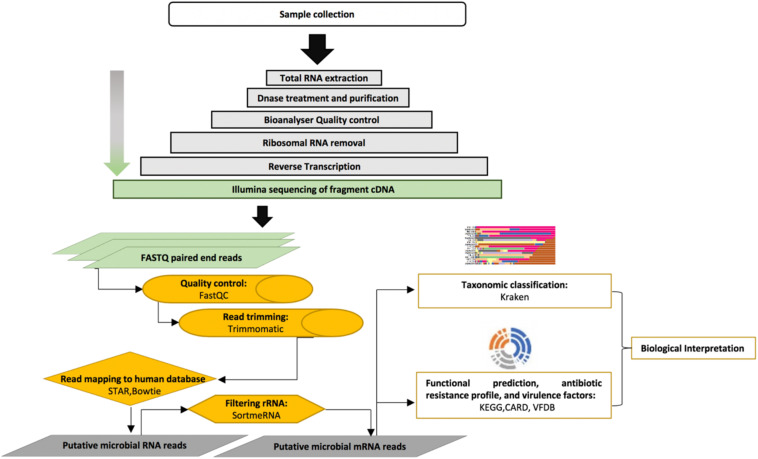
Overview of the metatranscriptome pipeline applied in this study.

Sequencing reads were passed through the FastQC quality control pipeline Version 0.11.7 (Andrews, 2010) to visualize their quality. Sequencing reads were trimmed and quality-filtered with Trimmomatic tool version 0.39 (LEADING:5, TRAILING:5, SLIDINGWINDOW = 4:15, MINLEN = 50) (Bolger et al., 2014). To filter human sequencing reads, trimmed reads were mapped to the human reference genome using ultrafast universal RNA-seq aligner (STAR v2.5.2a, human reference: GRCh37 genome including transcript annotation) (Dobin et al., 2013) and Burrows-Wheeler Alignment (BWA-mem version 0.7.15, human reference: GRCh38) (Li and Durbin, 2009). Putative non-human reads were then mapped to ribosome RNA databases (SILVA 16S, 23S, 28S, 18S rRNA, rfam 5S rRNA) using SortMeRNA v2.1 to identify and remove ribosomal RNA sequences (Kopylova et al., 2012). Putative bacterial mRNA was the target for further analyses (Figure 1).

### Bacterial Taxonomic Classification, Functional Profiling, DFI Resistome, and Expressed Virulence Factors

The taxonomic label assignment to sequencing reads was done using Kraken classifier v2 (an alignment program for assigning taxonomic labels to sequencing reads) (Wood and Salzberg, 2014). The database Kyoto Encyclopedia of Genes and Genomes (KEGG) (version 9 November 2016) was used to annotate the function of sequencing reads (Kanehisa and Goto, 2000) using DIAMOND v.0.99.

Visualization and statistical analysis were performed in Calypso 8.27 (Zakrzewski et al., 2016). ANOVA was used to determine if bacterial composition and function were significantly changed with infection severity.

The comprehensive antibiotic resistance database (CARD v3.0.1) (Jia et al., 2016) was used to identify bacterial transcripts carrying antibiotic resistance function (DFI resistome) using DIAMOND v.0.99. Antibiotic resistance genes in CARD were annotated as a present for a particular sample if at least 90% of the CARD reference sequence was covered by the metatranscriptome reads and with an average read fold of least 2.63.

The presence of bacterial virulence factors in metatranscriptome reads was evaluated using the Virulence Factor Database (VFDB) with at least 90% of coverage and an average read fold of 3.90 (Chen et al., 2016). The overview of the metatranscriptome pipeline used in this study is shown in Figure 1.

## Results

After the initial assessment of extracted RNA, 16 samples that had high integrity and quality RNA with either a mild (25%), moderate (31.25%), or severe infection (43.75%) were investigated in this study. The average age of patients was 60.80 ± 10.43 years (range from 34 to 77 years). Three patients were female, and 13 patients were male. Three patients (2 females and one male) had Type 1 diabetes, while remaining patients suffered from Type 2 diabetes. All patients suffered from peripheral neuropathy (100%) (Table 1).

**TABLE 1:** Demography of recruited patients in this study.

Patient ID	Age	Gender	Type of diabetes (1,2)	Duration of diabetes (Year)	Duration of ulcer (weeks) <2 (0), 2–6 (1), >6 (2)	PEDIS score 2 = Mild, 3 = Moderate, 4 = Severe
DFI109	51	Female	1	30	2	2
DFI111	51	Female	1	30	2	3
DFI112	68	Male	2	12	2	2
DFI113	54	Male	1	23	2	3
DFI114	72	Male	2	20	1	2
DFI117	34	Male	2	14	2	3
DFI119	61	Male	2	13	1	2
DFI121	61	Female	2	12	2	3
DFI126	54	Male	2	11	2	3
DFI153	65	Male	2	20	2	4
DFI155	62	Male	2	21	1	4
DFI160	59	Male	2	2	1	4
DFI161	68	Male	2	19	0	4
DFI166	71	Male	2	22	2	4
DFI167	77	Male	2	35	2	4
DFI171	64	Male	2	17	1	4

### Microbial Community Composition

**FIGURE 2:**
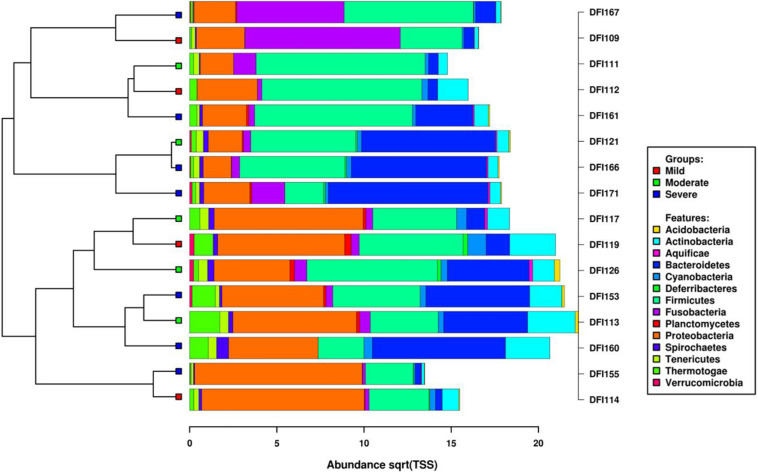
Clustered bar chart based on hierarchical clustering of the Bray–Curtis distances of the DFIs based on the taxonomic composition of active bacterial phyla as determined by metatranscriptome sequencing predicted using Kraken and visualized in Calypso. The horizontal axis is the square root abundances of the identified taxa.

RNAseq results in an average of 170 million paired-end reads (range 128 to 204 million) of 150 nucleotides per sample and on average 7.6 million paired-end reads per sample were taxonomically assigned to a bacterial taxon using Kraken. Fourteen bacterial phyla, 24 classes, 55 orders, 91 families, 109 genera, and 135 active species were identified in DFIs. Alignments of the sequencing reads using Kraken showed that the DFIs comprised of the phyla *Proteobacteria, Firmicutes, Bacteroidetes, Fusobacteria, Actinobacteria, Tenericutes, Cyanobacteria, Spirochetes, Thermotogae, Acidobacteria, Planctomycetes, Verrucomicrobia, Aquificae*, and *Deferribacteres* in descending order of the mean abundance in the samples. *Proteobacteria, Firmicutes*, and *Bacteroidetes* constituted the most abundant phyla. The abundance of individual taxa is visualized in Figure 2.

**FIGURE 3:**
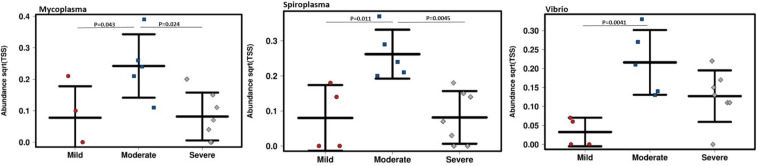
The genera *Mycoplasma*, *Spiroplasma*, and *Vibrio* significantly increased in moderate infections (*P* < 0.05). The vertical axis shows the square root abundances of each bacterial genus.

The genera *Proteus*, *Porphyromonas, Anaerococcus*, *Parvimonas*, and *Peptoniphilus* constituted the highest number of assigned sequencing reads in descending order. The genera *Mycoplasma, Spiroplasma*, and *Vibrio* were significantly abundant in moderate infections (*P* < 0.05, Figure 3).

**FIGURE 4:**
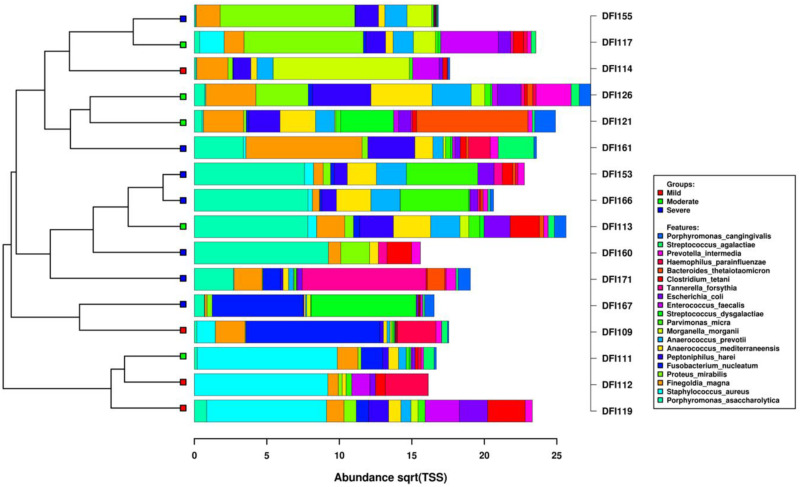
Clustered bar chart of the top 20 active bacterial species. The dendrogram was calculated using the hierarchical clustering of Bray–Curtis distances including the top 20 species assigned using Kraken. The horizontal axis is the square root abundance of the identified taxa.

Regardless of infection severity, *Proteus mirabilis*, *Porphyromonas asaccharolytica*, *Parvimonas micra*, *Anaerococcus mediterraneensis*, and *Peptoniphilus harei* had the highest number of assigned transcripts (Figure 4). Among samples with mild and moderate infections (*N* = 9), three samples were dominated by *Staphylococcus aureus*. *Porphyromonas asaccharolytica* was detected in all severe samples (*n* = 7) and dominant in four samples.

### Functional Annotations of the Transcripts in DFIs

Using similarity searches to the KEGG database, 132 functional pathways were identified in the DFIs. Six pathways were significantly changed in different infection severities (*P* < 0.05) (Table 2).

**TABLE 2:** Pathways significantly changed between infection severities (*P* < 0.05).

Pathway	Class	Increased in infection severity (PEDIS)
Lipoic acid metabolism	Metabolism of vitamins and cofactors	Mild
Two-component systems	Signal transduction	Mild
Bacterial invasion to epithelial cells	Bacterial infectious disease	Mild
Glycerolipid metabolism	Metabolism of lipid	Moderate
TCA cycle	Metabolism of carbohydrate	Moderate
Mismatch repair	Replication and repairing DNA	Severe

**FIGURE 5:**
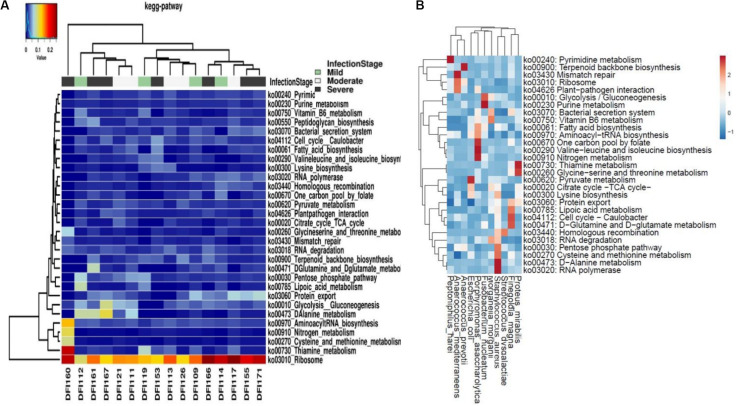
**(A)** Top 30 metabolic pathways predicted using the KEGG and visualized in Calypso. **(B)** Heatmap of KEGG pathways in highly transcriptionally active bacteria.

Pathways involved in ribosome and thiamine metabolism were the most abundant pathways (Figure 5A). A high number of ribosomal transcripts, particularly in *S. aureus*, *P. asaccharolytica*, and *Finegoldia magna*, may indicate high transcriptional activity and important metabolic roles of these species (Figure 5B).

### DFI Resistome Prediction

**FIGURE 6:**
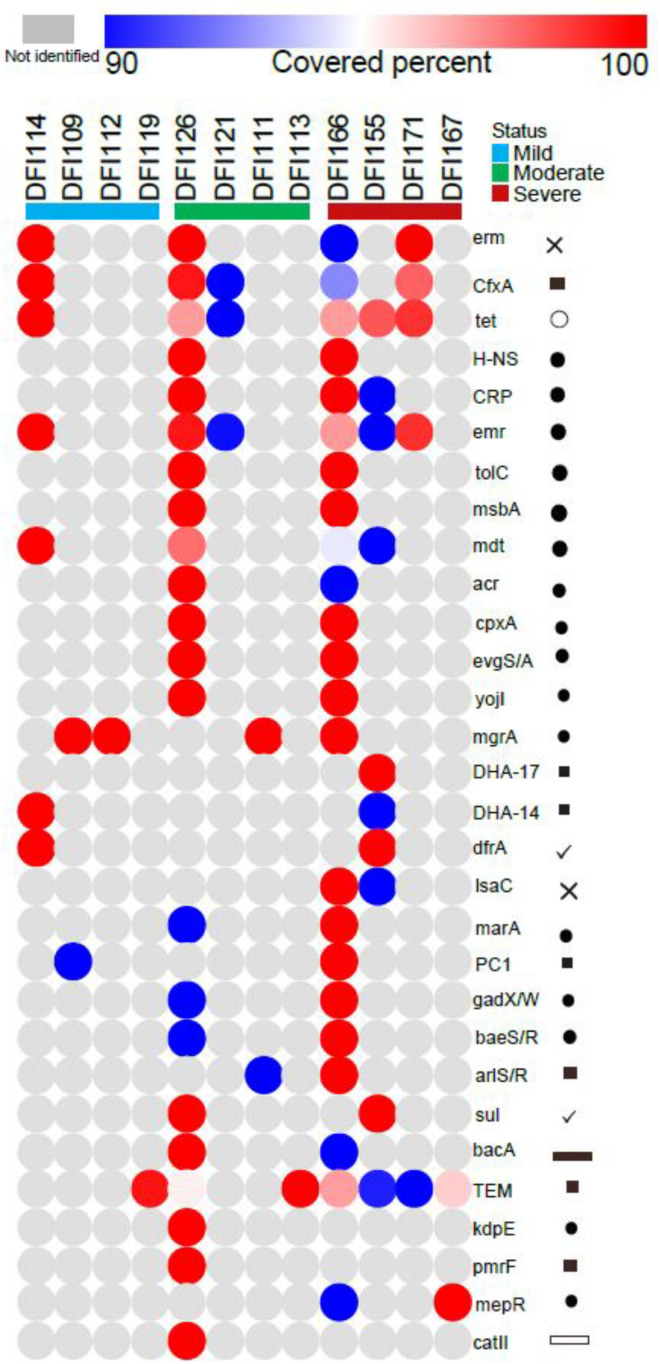
Detected antibiotic resistome in 12 DFI samples using the CARD. Antibiotic resistance genes encoding beta-lactam, macrolide, and tetracycline antibiotics were annotated mostly in moderate and severe infection. Genes related to multidrug efflux pumps/exporters and beta-lactam antibiotics were detected in 11 samples (×: macrolides, ■: β-lactam, ∘: polyketides, •: multidrug efflux pumps/exporters, ✓: sulfonamide, trimethoprim, 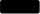: bacitracin, 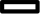: chloramphenicol). Circle color indicates the percentage coverage of the reference CARD sequence by the RNA-seq data (red/blue) or the absence in a sample (gray). Similar genes with similar functions were considered as one group. For instance, TEM-1, TEM-116, TEM-198, and TEM-157 were grouped as TEM.

Assignment of bacterial transcripts to the CARD database revealed the presence of 131 different genes directly or indirectly involved in antibiotic resistance mechanisms using the cut-off of >90% reference sequence coverage with the average read fold of at least 2.6 in 12 samples. No CARD feature was identified in four samples. The DFI resistome was mainly comprised of genes involved in the multidrug efflux pumps/exporters, resistance to beta-lactam, macrolide, and tetracycline antibiotics. TEM beta-lactamase resistance genes and genes involved in multidrug efflux pumps/exporters were detected in 11 samples. Tetracycline resistance gene (*tet*) and Erm methyltransferase were also detected in six and four samples, respectively. Furthermore, Gene *cfxA*, encoding a class A beta-lactamase was detected in five samples (Figure 6).

### Expressed Bacterial Virulence Factors in DFIs

**FIGURE 7:**
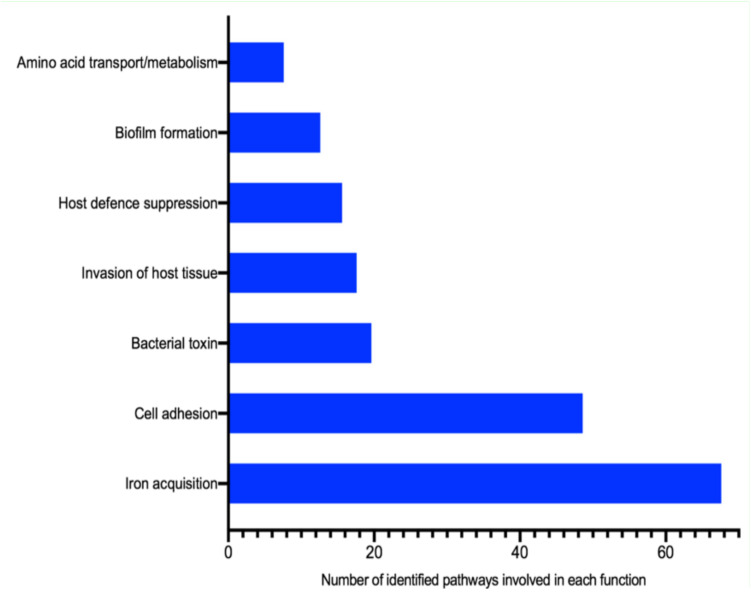
Identified bacterial virulence factors in DFIs using the VFDB.

The assignment of bacterial transcripts to the VFDB revealed the presence of 225 different mechanisms involved in bacterial pathogenicity using 90% coverage with the average read fold of at least 3.90 in seven samples. No VFDB feature was identified in nine samples. Overall, the most common virulence factors identified in DFIs comprised pathways involved in synthesizing and regulation of siderophores (iron-chelating molecules). The next common virulence factors were involved in bacterial cell-surface components (fimbria and flagellum) which facilitate adhesion, colonization, and movement of bacterial cells. Other pathways involved in the pathogenicity of pathogens are shown in Figure 7.

## Discussion

Diabetic foot infections are the most severe and costly complication of diabetes. With regards to the diabetic population, 50% of them are recognized to have ulcerated feet (Gupta and Kumar, 2019). Progression of DFIs to more complicated scenarios, such as minor and major amputation of a lower limb occurs every 30 s in the world which can influence the quality of life in many diabetic people worldwide^1^.

Identification of pathogenic bacteria is the first essential step to monitor and control the etiology of DFIs accurately (Sadeghpour Heravi et al., 2019). However, a large portion of bacterial species are not culturable using traditional methods (Suryaletha et al., 2018). While many human disorders have been linked to a shift in the bacterial composition/function (Nowicki et al., 2018), it is still unclear how this fluctuation may influence the development of DFIs.

Metatranscriptomics provides an extraordinary opportunity to systematically study bacterial communities (Zhao et al., 2016) including information about active bacterial compositions/functions which are necessary for the initiation and progression of the infection.

This study provides insights into the transcriptionally active bacterial population and its functionality in DFIs by means of a metatranscriptomic approach.

In this study, a high abundance of the transcripts assigned to the genera *Proteus*, *Porphyromonas*, *Anaerococcus, Parvimonas, Peptoniphilus, Prevotella, Fingoldia*, and *Streptococcus* were identified in DFIs. Taxonomic annotation revealed that bacterial pathogens in mild infection (PEDIS 2) were mainly *S. aureus*, *F. magna*, *Fusobacterium nucleatum*, and *Proteus mirabilis* which were commonly identified in DFIs (Gardiner et al., 2017). In moderate infections (PEDIS 3), *F. magna*, *P. harei*, *Morganella morganii*, and *Anaerococcus prevotii* were the most common assigned transcripts. In severe infections (PEDIS 4) *P. asaccharolytica*, *F. magna*, *Parvimonas mica*, *Anaerococcus mediterraneensis*, and *Proteus mirabilis* were predominant (Figure 4). These findings suggested that aerobic Gram-positive *cocci* such as *S. aureus* which has been the most described pathogen in DFIs were less common as the severity of infection increased. Transcripts assigned to the genera *Spiroplasma*, *Vibrio*, and *Mycoplasma* were significantly increased in moderate infections (*P* < 0.05), which indicated a major metabolic role of these genera in DFIs. The presence of transcripts assigned to both metabolically active aerobic (such as *S. aureus*, *Proteus mirabilis*, and *Escherichia coli*) and anaerobic microorganisms (such as *P. asaccharolytica*, *F. magna*, and *Fusobacterium nucleatum*) indicated the dual status of the bacterial lifestyle and the complexity of the bacterial communities in DFIs. The high prevalence of anaerobes and fastidious microorganisms (such as *Anaerococcus spp.* and *Peptoniphilus spp.*) may explain the inadequacy of culture-based methods in isolating the entire pathogens in DFIs.

Transcripts assigned to thiamin pathways dominated the DFIs. Since this pathway is a vital pathway in bacterial pathogenesis (Du et al., 2011; Costliow and Degnan, 2017) disrupting this pathway may lead to bacterial cell death and may suggest a novel target for treatment of DFIs (Schauer et al., 2009; Monjas et al., 2016). Also, the great abundance of ribosomal transcripts indicated the high translational activity of the bacterial population in our study (Bisanz et al., 2014) which may suggest the use and development of antibiotics that specifically target ribosomal function and subunits in bacterial pathogens.

The high abundance of bacterial transcripts assigned to a two-component system (TCS) in the mild stages of infection in this study (*P* < 0.05) (Table 2) and the importance of the TCS in bacterial survival may suggest a key role of the system as a new antimicrobial target. Furthermore, the high abundance of transcripts assigned to the bacterial pathway “invasion of epithelial cells” during mild infections (*P* < 0.05) may also suggest invading ability of pathogenic bacteria to enter epithelial cells and expand the infection in the early stages of infection (Ribet and Cossart et al., 2015; Table 2).

We have identified that most DFIs have complex metabolically active bacterial communities, thus understanding which bacteria contribute to the infective process could help to reduce the overuse of commonly prescribed antibiotics. Beta-lactam antibiotics are the most broadly prescribed antibiotics in the empirical therapy of DFIs. Extended-spectrum β-lactamase (ESBL) is the main resistance mechanism in Gram-negative bacteria, which results in multidrug resistance (MDR) pathogens. Based on our findings, genes conferring resistance to beta-lactams and genes involved in multidrug efflux pumps/exporters were detected in eleven samples. Tetracycline resistance genes (*tet*) and Erm methyltransferases were detected in six samples (Figure 6). Alarming levels of ESBL phenotypes, which were resistant to many classes of commonly prescribed antibiotics such as penicillins and cephalosporins have been reported in many different studies which were concordant with our findings suggesting more precaution in the prescription of these antibiotics in the treatment of DFIs (Motta et al., 2003; Turhan et al., 2013).

Also, the iron acquisition system (synthesize and regulation of siderophores) was the most common mechanism involved in the pathogenicity of bacterial cells in DFIs. Pathways involved in the synthesis and regulation of cell-surface components associated with adhesion, colonization, and movement of bacterial cells were the next common virulence factors. These virulence factors may help bacteria compete for scares resources and survive the host wound proteases. This may explain the importance of the aforementioned systems in the pathogenicity of bacterial cells and targeting these factors to prevent bacterial cells more effectively (Figure 7).

However, obtaining RNA with high RIN from infected clinical samples suitable for RNA sequencing approach is very challenging, after the initial assessment of the extracted RNA, we were limited to low sample size as RNA in some infected tissue may have already been degraded *in situ*.

RNA sequencing is very costly as well and needs deep sequencing (average of 170 million reads per sample in this study) to be able to capture enough bacterial RNA signals as > 95% of sequencing reads belonging to human RNA reads in clinical samples. However, recent advances in the genomics field over the past quarter-century have led to considerable reductions in the sequencing costs. Since these methods are becoming frontlines in medical laboratories, it may be projected that this reduction to be continued in order to influence the scale and scope of research projects investigating genomic aspects of bacterial communities (Marshall et al., 2017).

To the best of our knowledge, this is the first study to apply an RNA-sequencing technique to profile the active bacterial communities in DFIs. Our findings can help to identify the composition and function of bacterial communities in DFIs. However, further experimental research is needed to evaluate the pathogenicity of the identified bacterial species and the application of detected pathways in the treatment of DFIs.

## Conclusion

Based on our findings, treatment strategies targeting a single species or specific bacterial pathways might be ineffective in the treatment of DFIs, and a multifaceted therapy is required. Abundant pathogens and pathways identified in this study may be possible biomarkers to prevent infection in the future.

## Data Availability Statement

The datasets presented in this study can be found in online repositories. The names of the repository/repositories and accession number(s) can be found at: https://www.ncbi.nlm.nih.gov/, PRJNA563930.

## Ethics Statement

This study was approved by the South West Sydney Local Health District Research and Ethics Committee (HREC/14/LPOOL/487, SSA/14/LPOOL/489) and Macquarie University Human Ethics Committee (Reference No. 5201500839). The patients/participants provided their written informed consent to participate in this study.

## Author Contributions

FH conducted the laboratory experiments and data analysis and wrote the manuscript. MZ analyzed the data, and reviewed and commented on the manuscript. KV and MM reviewed and commented on the manuscript. HH designed the project, monitored the laboratory experiments, and reviewed and commented on the manuscript. All the authors read the final manuscript and consented the publication.

## Conflict of Interest

The authors declare that the research was conducted in the absence of any commercial or financial relationships that could be construed as a potential conflict of interest.

## Footnotes

1 Https://Idf.Org/Our-Activities/Care-Prevention/Diabetic-Foot.Html/ Accessed:2020
